# Artificial intelligence in pituitary surgery: the path to clinical solutions

**DOI:** 10.1530/ERC-26-0104

**Published:** 2026-08-20

**Authors:** George Hudson, Danyal Z Khan, Stephanie E Baldeweg, Hani J Marcus

**Affiliations:** ^1^ Victor Horsley Department of Neurosurgery, National Hospital for Neurology and Neurosurgery, London, UK; ^2^ UCL Queen Square Institute of Neurology, London, UK; ^3^ Department of Endocrinology, UCLH, London, UK; ^4^ Department of Medicine, UCL, London, UK; ^5^ Panda Surgical Ltd, London, UK

**Keywords:** pituitary, adenoma, neuroendocrine tumours

## Abstract

Artificial intelligence (AI) is rapidly moving from conceptual innovation to high-performing algorithms across the pituitary patient pathway, promising benefits to patients, endocrinologists and surgeons alike. Pre-operatively, machine learning applied to facial imaging and natural language processing of electronic health records show potential for earlier identification of pituitary adenomas and timelier referral from primary providers to a specialist endocrinologist. Intraoperatively, computer vision systems have already augmented surgical training but have the potential to become integrated real-time decision support systems. Post-operatively, predictive models may help to forecast complications and longer-term remission, endocrinological outcomes or recurrence. Across these domains, the emergence of large, multimodal datasets, which integrate clinical text, endocrinological investigations, radiological imaging, and intraoperative video, promises further AI improvements, yet the impact of AI on real-world clinical decision-making is less clear and depends as much on implementation processes and clinicians themselves. This review provides an overview of AI technologies for pituitary patients and explores some of the challenges translating them to clinical practice.

## Introduction

Over the past five years, the care of patients with pituitary disease has become progressively digitised, with routine care generating detailed data across electronic health records, imaging and operative video. These multimodal datasets have improved the performance of existing machine learning (ML) techniques, while creating new potential for artificial intelligence (AI)-guided priority setting across diagnosis, operative workflows and follow-up.

Pre-operatively, functioning adenomas may cause syndromes such as Cushing’s disease or acromegaly, while macroadenomas may impinge the optic chiasm, producing visual field loss (1). The insidious onset of these symptoms means that diagnosis is often protracted, with progressive endocrinological morbidity and more complex surgical cases. For surgery itself, the endoscopic transsphenoidal approach is now commonplace but has a recognised learning curve (2), requiring careful planning and fine movements to avoid neurovascular injury. Post-operatively, electrolyte and hormonal dysregulation are difficult to predict, necessitating specialist endocrine input to manage arginine vasopressin deficiency (AVP-D), syndrome of inappropriate anti-diuretic hormone secretion (SIADH) and hypopituitarism. Over the long term, recurrence risk is unique to each patient requiring a personalised approach.

In this space, large, multimodal datasets and advances in cloud-based computational infrastructure have accelerated the development and adoption of AI technologies. Large language models (LLMs) learn the contextual structure of language and can process clinical documentation, extract relevant information and generate structured reports. More recently, the availability of labelled image and video datasets has enabled rapid progress in computer vision, a branch of AI that allows computers to analyse visual data, identify meaningful patterns and generate image outputs in real time.

**Figure 1:**
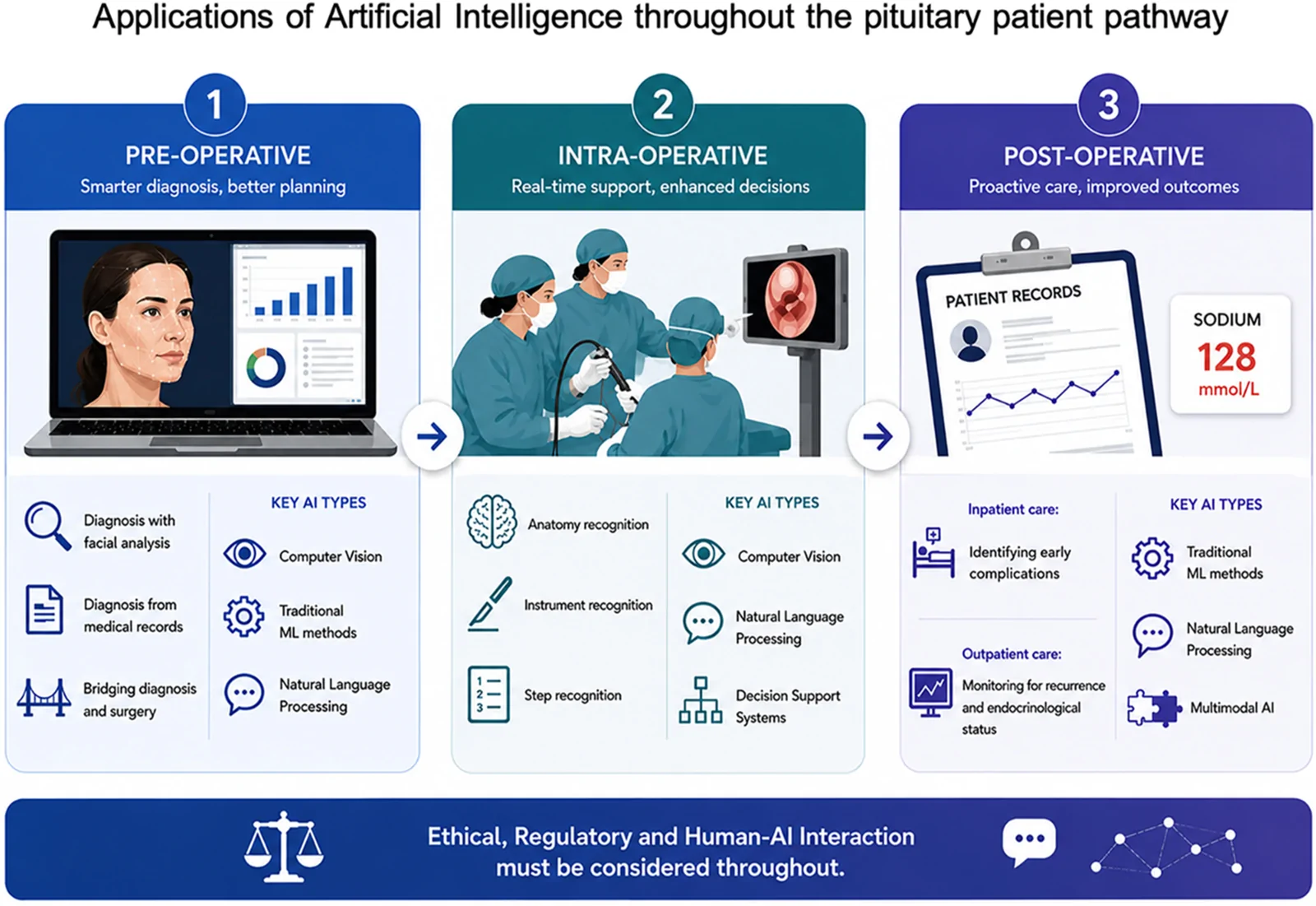
AI has the potential to shape pituitary patient care across the patient pathway.

In our previous review, we examined the potential of AI to supplement clinical practice in the care of pituitary patients (3). Priority setting partnerships have since identified important areas of pituitary research (4), and modern, multimodal datasets have rapidly expanded. Nonetheless, the success of these technologies is highly dependent on human factors, such as culture and workflows, which affect their adoption into routine care. In the current review, we update our prior assessment – evaluating not only the advancement of AI algorithms and data but also the potential for AI to shape endocrinological and oncological decision-making in key areas across the patient pathway (Fig. 1).

## Pre-operative applications of AI

Patients with pituitary adenomas often experience significant delays in diagnosis (5), allowing tumours to enlarge and disrupt endocrinological axes. This provides time for tumour invasion into the adjacent cavernous sinus or suprasellar space, which affects the ability to achieve resection in the operating room (6). Earlier AI-facilitated diagnosis, using a variety of different approaches as described in Table 1, has strong potential to transform both endocrinological and surgical outcomes.

**Table 1:** Description of common terminology.

Terminology	Description
Artificial intelligence (AI)	A computer system capable of mimicking human intelligence across tasks such as reasoning, learning and decision-making
Machine learning (ML)	A subset of AI focused on algorithms that learn patterns from data by optimising model parameters to minimise error on prediction or classification tasks
Deep neural network	A machine learning model consisting of multiple layers of artificial neurons that transform inputs through weighted connections, typically learnt using techniques such as gradient descent and backpropagation, into feature representations
Foundation model	A large-scale neural network trained on massive, diverse datasets to learn general-purpose representations that can later be adapted to downstream tasks
Transfer learning	A machine learning technique where a model trained on one task or dataset reuses its learned representations and weights as a starting point for a different but related task, improving performance and reducing training time and data requirements
Natural language processing (NLP)	A field of AI focussed on enabling computers to understand, interpret and generate human language
Large language model (LLM)	A deep neural network, typically based on transformer architecture, trained on massive text data to learn contextual representations of language
Transformer	A neural network architecture designed for sequence modelling that uses self-attention mechanisms to compute relationships between all elements in a sequence, allowing the model to focus on the most relevant information
Computer vision	A field of AI that enables computers to interpret and understand visual information from images and videos
Convolutional neural network (CNN)	A specialised deep neural network designed for processing data such as images, using convolutional layers with learnable filters to detect spatial features, such as edges, textures and object structures

### Diagnosis with facial analysis

For functioning adenomas, one of the first patient signs of disease may be change in appearance. Geometric node-based analysis and convolutional neural networks (CNNs), two computer vision techniques, are accurate tools in diagnosing facial images of Cushing’s disease and acromegaly (7). Geometric node-based analysis quantifies the spatial relationships between pre-defined facial landmarks, such as the eyes, nose and jawline. Complementing this, CNNs analyse facial images hierarchically by processing small image patches across multiple layers and resolutions, enabling the extraction of visual features including texture, contour and soft tissue prominence. Together, these methods can identify characteristic pathological facial signatures associated with hormone excess states. In the past, studies deploying these techniques demonstrated the feasibility of AI screening but relied on curated, retrospective data banks. To detect cases in the community, computer vision algorithms must be robust to the lighting changes, variable photo angles and local demographics, which can all affect facial appearance.

In recent years, the emergence of foundation AI models trained on large datasets of generic images has advanced image analysis. Transfer learning leverages these pre-trained models with broadly learned visual features and fine-tunes them for specialised clinical tasks using comparatively small datasets. For facial analysis in endocrinological pathology, such strategies achieve high diagnostic performance using sparse datasets of under 200 patients (8) or using images that are not perfectly forward-facing (9). Information may also be streamed to different types of ML algorithms, for example using traditional methods for geometric analysis, and CNNs for texture analysis where context matters more (10). In turn, these new pipeline architectures improve the accuracy of ML in real-world patients.

Clinically, there has also been a shift away from simple classification and towards changing practice. AI has been trained to recognise acromegaly years before diagnosis, minimising tumour growth and making achieving cure potentially easier (11). Comparisons to large panels of endocrinologist experts have shown non-inferior performance representing stronger evidence of efficacy (9). In this way, today’s AI systems are more relevant to the clinician managing pituitary pathology by allowing them to identify disease sooner.

### Diagnosis from medical records

Alongside personal photography, the use of chatbots has boomed in recent years and the development of natural language processing (NLP) offers another opportunity to make an early diagnosis. While hospital systems regularly use clinical codes to measure activity, these rely on user inputs and may not always capture the complexity of an encounter. NLP is the use of AI to interpret and replicate patterns of language and offers the opportunity to comb through unstructured, free-text information and provide dynamic summaries automatically. Nonetheless, in previous work, NLP focused on hospital-led information extraction from free text using dictionary lookups (12). This approach allowed clinical records to be queried but remained fundamentally rigid.

Transformer neural network structures have since paved the way for the emergence of LLMs. These use a series of attention blocks to perform matrix multiplications and encode contextual meaning through the relationships between words. They then use multi-layer perceptron blocks to perform operations where different parts of the sentence can contribute differently to the end output, aiming to extract higher-level relationships. Cloud-based computing is fundamental to these models – centralising graphics processing units (GPUs), which can perform several calculations in parallel. This means models can be trained and accessed in separate geographical locations for real-time use in high-dimensional spaces from the patient’s own phone.

To date, LLMs have been used to determine the cause of death from post-mortem CT reports (13) or propose differentials from complex case histories (14). Yet, the development of products such as ChatGPT Health opens the door to daily symptom tracking – especially useful for subtle endocrinological symptoms. For example, chatbots may suggest a differential diagnosis based on endocrinological symptoms, provide guidance for further workup and prompt to consider referral to an endocrinologist. As with facial imaging, LLMs have seen more alignment with clinical needs too. LLMs can now be coupled with retrieval-augmented generation (RAG) to draw on institution-specific protocols to suggest actionable MRI sequences (15). Once the MRI has been performed, LLMs may then play a role in report interpretation (16). In this way, NLP has evolved from a backend data mining tool into a front-line clinical interface for both the patient and the endocrinologist for diagnostic workup.

### Bridging diagnosis and surgery

Once diagnosed, some patients are medically managed in the first instance by an endocrinologist. ML models that use radiological features from MRI as predictors (radiomics) are relatively accurate in predicting response to somatostatin analogues in acromegaly (17) and dopamine agonists in prolactinomas (18). At present, however, these methods have not been compared to expert predictions and have not been implemented prospectively as a tool to streamline care without trial of medical management first. For patients with operable disease, there may also be a wait before intervention, which could limit the real-world impact of sub-streaming patients for early surgery.

While patients wait for intervention, AI may alternatively play a role in tackling their questions, making patients more informed and empowering them to seek out specific treatment options. For example, LLMs can provide responses to pituitary-related questions, which are highly reliable and consistent (19). Likewise, LLMs can establish patient sentiment and classify concerns thematically (20), potentially supporting more personalised discussions about operative risks and consent. More broadly, patient interactions with LLMs may provide insight into how their pathology affects their mental health, serving to identify new ways to support patients throughout their treatment – a key research priority. From a clinician’s perspective, AI chatbots may help to risk-stratify patients and guide perioperative management. When embedded with institutional perioperative protocols, LLMs can provide highly accurate advice, in many cases even expediting care (21). In turn, this can change practice: minimising delays on admission and making more efficient use of resources.

However, for pituitary patients, these pre-operative systems remain to be implemented into clinical practice, limited by lack of clear benchmarks and through ambiguity over who funds their roll-out. For individual general practitioners, return on investment is challenging given the relative rarity of pituitary adenomas. This may mean these systems find a more comfortable home in the consumer market where their translation will be delayed not by clinical impact, but by consumer appetite for a broader suite of diagnostic tools.

Moreover, new challenges may emerge as these tools integrate into the patient pathway. In pituitary disease, hormone measurements vary by time of day, and the endocrinologist must manage biochemistry and symptomatology on a background of complex medical histories. In this setting, AI-assisted advice may produce inaccurate or biased answers. Under traditional fault-based liability frameworks, patients who have experienced harm must establish a breach of a duty of care with clear causation; however, AI black-box systems make it difficult to attribute blame and harm may still occur even when all human players behaved appropriately (22). Indeed, patients may not even realise the agency of these platforms in their care. Tackling these issues relies on multi-stakeholder planning and shared responsibility to effectively translate the benefits of diagnostic AI, without losing patient trust.

## Intraoperative applications of AI

For patients amenable to surgery, pituitary surgeons operate with limited manoeuvrability around delicate neurovascular structures. To date, AI tools that can establish operative steps, recognise instrument selection and delineate intraoperative anatomy have been developed. These tools have already shown good utility outside the operating theatre but may soon assist in intraoperative decision-making to improve surgical and endocrinological outcomes.

### Task-specific AI

Endoscopic pituitary surgery has clear operative steps, adherence to which can minimise delay and maximise safety across the operative workflow (23). These steps can be recognised using computer vision tools and subsequently fed into recurrent neural networks, which incorporate preceding frame predictions for better temporal consistency (24). This type of analysis has even been combined with ML methods to estimate remaining operating time (25).

For the operation itself, endoscopic surgery typically involves rapidly revolving instruments, each used for a specific part of the operation. Computer vision can segment these instruments as they appear. Using a ResNet50 encoder and a DeepLabV3 decoder, AI models can capture features at multiple scales without losing image resolution, which is useful because surgical instruments appear at different sizes as they move in and out of view. These features can then be fed into an advanced object-tracking algorithm that follows them across frames and compensates for camera movement, achieving accurate tracking (26).

Complementing instrument recognition, AI can highlight patient anatomy. During endoscopic pituitary surgery, there is a risk of carotid artery injury as these vessels pass through the cavernous sinus, as well as optic nerve injury as the nerves traverse the suprasellar space. Previously, intraoperative MRI and ultrasound were proposed to reduce the risk of damaging these structures, but these technologies disrupt surgical workflow, requiring pauses for equipment placement. To improve navigation, CNN architectures, such as HRNet, preserve high-resolution representations while simultaneously learning lower-resolution features, allowing both fine anatomical details and broader structural context to be captured. Modern methods also use Vision Transformers (ViTs), which adapt the transformer architecture to images by splitting an image into patches and then using attention blocks to capture wider context across these patches. The resulting features generate overlays of key anatomical structures, such as the carotid artery and optic nerves, from the indentations they create in the sphenoid (27).

### Clinical translation

For the operating surgeon, combinations of these AI solutions can support training and intraoperative decision-making. For example, operative workflow analysis, where AI decodes the operative steps, has been successful when combined with a dedicated coaching programme, allowing trainees to review their prior operations and navigate to key parts with ease. Surgeons completing such coaching showed significant improvements in their real-world operative performance after 6 months (28).

Likewise, instrument tracking has merits as a surgical training tool. Metrics based on instrument movement economy and visibility time have been linked to surgical performance, allowing segregation of experts and novices (26). In the future, this may help trainees to reflect on their instrument tissue handling, fast-forwarding their learning curve.

Going further, AI anatomical recognition of the sella augments surgical performance in real time outside the operating theatre. When identifying the sella on still images, more than half of the surgeons changed their mind after seeing the AI’s outline as a recommendation, with a resulting significant improvement in performance (29). This was particularly the case for medical students whose DICE scores, measuring overlap of the user’s planned resection with the true sella, improved by over 12 percentage points after using the computer vision tool. Simulation studies, yet unpublished, have similarly found that use of a computer vision AI as a decision support system can be effective in levelling the performance gap between experts and novices.

As these tools transition to clinical practice, the goal is for AI to act as an expert assistant, offering context-aware recommendations: vision-language AI systems represent a new class of intraoperative AI support for this purpose (30). These systems, such as the model PitVQA-Net, leverage an AI architecture that employs computer vision techniques to extract spatial features from endoscopic video, which are then fused with semantic features generated by transformers to allow users to ask questions about the stage of the operation. Another system, SurgicalVLM-Agent, extends this framework – incorporating PitVQA-Net as a module within a larger architecture to provide temporal context (31). This allows the system to become an intraoperative chatbot, translating the image on the screen into words and then understanding how this fits with the overall operative objective.

These aspirations aside, today’s computer vision systems are only just at the point of clinical translation, with first in-human trials of pituitary computer vision tools currently ongoing (32). As such, little is known about their safety, reliability and impact on real operative outcomes. Despite this, frameworks such as the IDEAL and DECIDE-AI guidelines provide instructions as to how early clinical development of these tools should proceed to allow others to replicate the findings. These guidelines further emphasise the importance of considering multiple stakeholders from early in the development process and highlight how user trust in these tools should be appropriately calibrated to ensure safe deployment (33). By adhering to these frameworks, the path towards AI in the operating room has been slow but has stronger promise for reaping the rewards at clinical translation.

## Post-operative applications of AI

In patients undergoing an operation, electrolyte and hormonal imbalances and residual tumour following surgery are common challenges. In this space, the goal of AI is to forewarn the clinician and provide actionable information to guide management as an inpatient or outpatient. For both endocrinologists and surgeons, this is an active research priority.

### Inpatient care: identifying early complications

In contrast to pre- and intraoperative AI domains, progress in post-operative AI has been more constrained. Following pituitary surgery, post-operative sodium imbalances are a major source of prolonged length of stay and increased morbidity. These imbalances are challenging to predict using traditional ML techniques. Early ML methods often included variables that were difficult to collate in real practice and produced predictions across timeframes which were not useful (34). As a result, endocrinologists were reluctant to adopt these predictive tools because the outputs could not effectively guide fluid restriction decisions or influence discharge planning.

Subsequent work used multimodal and prospectively validated models: radiological features, such as pituitary stalk deviation, have been incorporated into hyponatraemia prediction (35), visual outcome has been predicted with accuracies approaching 89% (36), and cerebrospinal fluid (CSF) leak risk has been modelled in large cohorts (37). Despite these accuracy improvements, complications are relatively uncommon, and unlike pre-operative diagnosis, there is no consensus on predictor variables, with intraoperative video data not included. This has already shifted emphasis away from headline accuracy and towards how model outputs adjust pretest probability and clinical decision-making. This has revealed only modest benefit (38). Moreover, the default post-operative protocol is already cautious, making it challenging for an uncertain AI to have a meaningful impact, ultimately limiting post-operative predictive AI to a proof of concept without further clinical roll-out.

Against this backdrop, NLP has emerged as a useful post-operative tool. Rather than forecasting rare events, NLP can synthesise fragmented information contained in medical records. NLP methods, such as CogStack, can already predict complications such as CSF leaks after vestibular schwannoma surgery (39), or intensive care admission in a wider neurosurgical cohort (40). The ability of transformer-based AI to process contextual dependencies may in the future identify patterns previously unbeknownst to clinicians governing electrolyte imbalance. For example, there is potential for computer vision AI to process intraoperative video and extract patterns of surgical manipulation that may predict dysnatraemia. Modern multimodal frameworks, including systems such as Google MedGemma, may also take this a step further. While previous ML approaches relied on pre-defined predictor variables selected for specific outcomes, transformer-based architectures that use attention-based processes to look at a wider context can integrate heterogeneous clinical data simultaneously. Therefore, rather than functioning solely as endpoint-specific predictors, the multimodal models of the future could provide a broader peri-operative sense check before discharge, evaluating possible complications in a cross-cutting way.

### Outpatient care: monitoring for recurrence and endocrinological status

In the long term, endocrinological status, residual tumour, and long-term recurrence represent some of the most clinically challenging aspects of pituitary outpatient care. In the previous review, AI-driven deep learning reconstruction (DLR) was highlighted as a means of improving post-operative MRI, with increased signal-to-noise ratio and enhanced sensitivity for detecting residual adenoma (41). ML has also been used for predicting recurrence or remission of acromegaly or Cushing’s disease (42, 43).

More recently, studies with follow-up periods extending to 15 years have used standard ML methods to model disease progression (44). Likewise, ML can predict medium- and long-term hypopituitarism in patients with non-functioning pituitary adenomas (45). In this study, rates of panhypopituitarism varied across a 10-year period, illustrating the challenge faced by endocrinologists when deciding interval follow-up, yet ML was able to accurately predict panhypopituitarism over this timeframe. Moreover, feature importance analysis identified transition points whereby pre-operative endocrinology profiles were informative to 1 year post-operatively, but predictions at the five- and ten-year mark were more guided by profiles taken 1 year post-operatively. As such, ML may also help to plan the timing of benchmark blood tests.

At present, long-term recurrence predictive AI is not routinely used in clinical practice and remains constrained by the same structural problem that limits complication modelling: signals are noisy, delayed and rarely change practice. This is reflected in meta-analysis findings, which show logistic regression sometimes performs as well as or better than more complex approaches, raising questions about the value for money provided by advanced AI in this space (46, 47). For example, in studies evaluating clinical indicators of recurrence, strong predictors include modified Knosp grade and resection extent – both already intrinsically linked to residual disease. In studies employing radiomics, predictors included pre-operative tumour volume doubling time – again intrinsically linked to tumour growth. The inclusion of these predictors, already intuitively understood by the multidisciplinary team without AI assistance, casts doubt on the value added by complex computational AI models.

There is also the question of how the outputs of these predictive platforms are used. For functioning adenomas, even the smallest uncertainty in the AI would demand endocrinological testing. More useful therefore would be information relating to when remission may be observed to help guide the decision to return to theatre. Likewise, AI, which can integrate genomics, histopathology and imaging, may help in long-term monitoring programmes. However, this actionable information is not yet targeted by AI algorithms. Thus, unlike pre- and intraoperative AI, which have undergone years of pipeline optimisation, AI predictions of post-operative events are not yet accurate nor granular enough to guide decision-making. For health systems with limited slack and a tight public purse, investing in such predictive AI is currently hard to justify.

## Conclusion

In summary, AI in pituitary patient care has evolved from isolated predictive models into algorithms capable of more appropriately addressing the challenges faced by pituitary patients. While the high accuracy of individual algorithms was previously established, the increasing availability of data has made AI outputs more robust and pipeline architectures have improved their efficiency.

There has also been a parallel shift towards technologies designed to integrate with existing workflows. LLMs offer instantaneous decision support, while computer vision can inform operative strategy itself. Post-operatively, predicting sodium imbalances, cure and hypopituitarism has the potential to shape management and follow-up frequency in the community, facilitating more effective use of clinical resources.

Despite this progress, AI is scarcely being used routinely in the clinical care of pituitary patients. Widespread adoption depends on evidence demonstrating meaningful improvements in patient outcomes and adjusted clinical decision-making – without this, the economic and ethical justification for implementation remains limited. Some areas are closer to translation than others: intraoperatively, computer vision systems have been introduced into theatres in first in-human studies (32); meanwhile, AI focussing on pre-operative early diagnosis or post-operative follow-up has not yet been integrated convincingly into clinical workflows.

Over the coming decade, as AI becomes embedded within everyday healthcare, the cultural and philosophical shifts needed to overcome these barriers may be driven as much by patient expectation as by technological progress. Frustration with delayed patient pathways and growing awareness of AI in the wider community may generate change and encourage clinicians to consider how AI can best partner with existing practice. In turn, this may pave the way for real clinical translation of AI for pituitary patients.

## Declaration of interest

Prof. Hani J Marcus is an employee of, and holds equity in, Panda Surgical. The other authors declare that there is no conflict of interest that could be perceived as prejudicing the impartiality of the work reported.

## Funding

This work did not receive any specific grant from any funding agency in the public, commercial or not-for-profit sector. Mr Danyal Z Khan is supported, more generally, by an NIHR Doctoral Fellowship and Google PhD Fellowship. Prof. Hani J Marcus is supported by the NIHR ULCH/UCL Biomedical Research Centre.
